# A novel variant in *MYLK* causes thoracic aortic dissections: genotypic and phenotypic description

**DOI:** 10.1186/s12881-016-0326-y

**Published:** 2016-09-01

**Authors:** Matias Hannuksela, Eva-Lena Stattin, Joakim Klar, Adam Ameur, Bengt Johansson, Karen Sörensen, Bo Carlberg

**Affiliations:** 1 Department of Surgical and Perioperative Sciences, Heart Centre, Umeå University, 901 85 Umeå, Sweden; 2 Department of Immunology, Genetics and Pathology, Science for Life Laboratory, Uppsala University, Uppsala, Sweden; 3 Department of Medical Biosciences, Medical and Clinical Genetics, Umeå University, Umeå, Sweden; 4 Department of Public Health and Clinical Medicine, Umeå University, Umeå, Sweden; 5 Department of Radiation Sciences, Umeå University Hospital, Umeå, Sweden

**Keywords:** Thoracic aorta, Aortic dissection, Gene mutation, *MYLK*

## Abstract

**Background:**

Mutations in *MYLK* cause non-syndromic familial thoracic aortic aneurysms and dissections (FTAAD). Very little is known about the phenotype of affected families. We sought to characterize the aortic disease and the presence of other vascular abnormalities in FTAAD caused by a deletion in *MYLK* and to compare thoracic aortic diameter and stiffness in mutation carriers and non-carriers.

**Methods:**

We studied FTAAD in a 5-generation family that included 19 living members. Exome sequencing was performed to identify the underlying gene defect. Aortic elastic properties measured by TTE, MRI and pulse wave velocity were then compared between mutation carriers and non-carriers.

**Results:**

Exome sequencing led to the identification of a 2-bp deletion in *MYLK* (c3272_3273del, p.Ser1091*) that led to a premature stop codon and nonsense-mediated decay. Eleven people were mutation carriers and eight people were non-carriers. Five aortic ruptures or dissections occurred in this family, with two survivors. There were no differences in aortic diameter or stiffness between carriers and non-carriers of the mutation.

**Conclusions:**

Individuals carrying this deletion in *MYLK* have a high risk of presenting with an acute aortic dissection or rupture. Aortic events occur over a wide range of ages and are not always preceded by obvious aortic dilatation. Aortic elastic properties do not differ between carriers and non-carriers of this mutation, rendering it uncertain whether and when carriers should undergo elective prophylactic surgery.

## Background

Knowledge of the genetic background of thoracic aortic aneurysms and dissections has increased remarkably in recent years. An estimated 20 % of patients with these aneurysms and dissections have a family history of the disease [1]. Thoracic aortic aneurysms are usually asymptomatic until a catastrophic complication such as a dissection or rupture occurs. Dissections are associated with high mortality and morbidity; many of the patients with type A dissection die before they reach a hospital, and an additional 25 % die while hospitalized [2–4]. Therefore, it is important to identify patients at risk for dissection and to monitor aortic dimensions in order to provide elective prophylactic intervention when needed.

The underlying genetic cause of thoracic aortic aneurysms and dissections must be taken into account when deciding whether prophylactic operation is appropriate. For patients with the sporadic form of the disease, surgery is recommended when the diameter of the ascending aorta (AoA) reaches 5.0–5.5 cm. In familial thoracic aortic aneurysms and dissection (FTAAD) dissection may occur at smaller diameters or even in aortas without obvious dilation [1].

Several genes have been associated with FTAAD [5]. The molecular mechanism of aortic dissection may result from dysfunction in the contractile apparatus of smooth muscle cells (SMCs) in the aortic wall (mutations in *ACTA2, MYH11, MYLK*), by disturbances in TGF-β signaling (mutations in *TGFβ2, TGFβ3, TGFβR1, TGFβR2, SMAD3*) or by dysfunction of the extracellular matrix (mutations in *FBN1, COL3A1*) [6].

In 2010, mutations in the gene encoding myosin light chain kinase gene (*MYLK*; protein MLCK; chromosome 3q21.1) were shown to cause aortic dissections [7]. Two novel mutations (p.R1480* and p.S1759P) were identified as co-segregating with the disease in two families with FTAAD. All affected family members presented with acute aortic dissections at various ages. Notably, aortic dissection occurred with little or no aortic enlargement. Nonetheless, the phenotypic features of *MYLK* mutations have only been briefly described in one previous publication [7].

MLCK, which is highly expressed in SMCs, phosphorylates the regulatory light chain of myosin to initiate SMC contraction. A previous study has demonstrated that the pathogenic *MYLK* variant p.R1480* was a nonsense mutation expected to cause nonsense-mediated decay and a truncated protein lacking the kinase and calmodulin binding domains, and the p.S1759P alters calmodulin binding sequence, thus abrogating MLCK function [7]. Altered gene expression and medial degeneration of the aorta were observed in mice with SMC-specific knockdown of *MYLK* [7].

Here, we identified a 5-generation family with aortic dissection that was inherited in an autosomal dominant fashion. Exome sequencing revealed a disease-causing 2-bp deletion in *MYLK* (c.3272_3273del, p.Ser1091*). We also investigated the genetic and phenotypic characteristics of this family.

## Methods

### Subjects

**Fig. 1:**
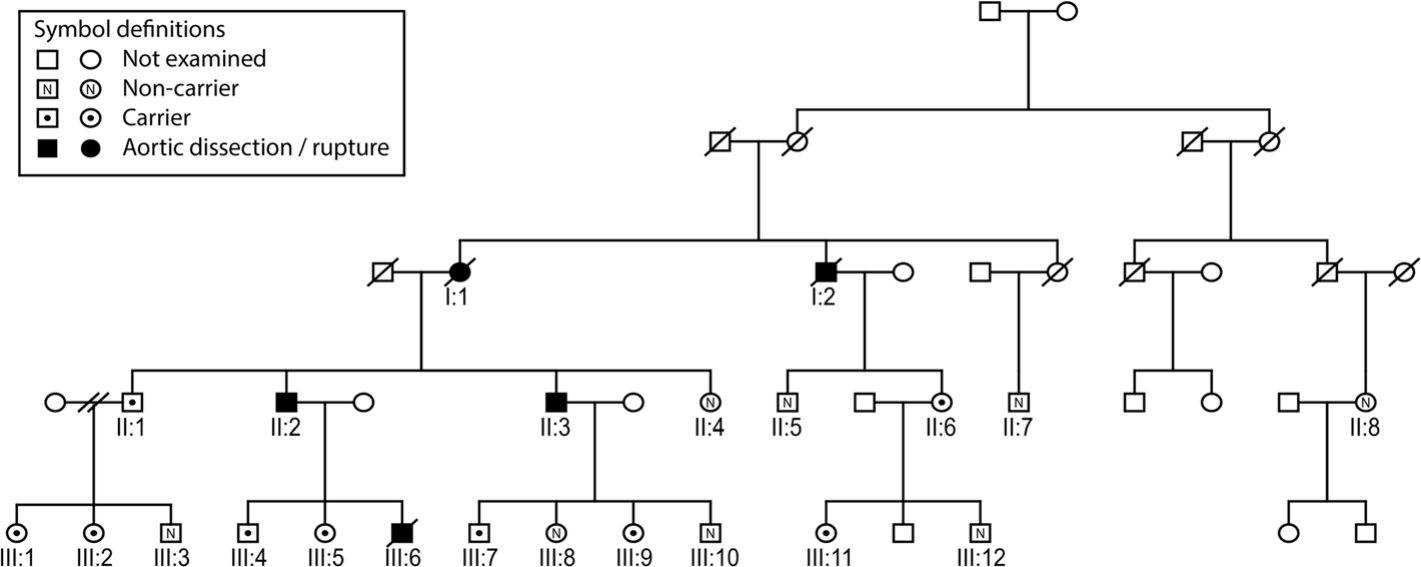
Pedigree of the investigated family

A large family (Fig. 1) with aortic dissection inherited in an autosomal dominant fashion was referred to the Centre for Cardiovascular Genetics of Umeå University Hospital, Sweden for clinical and genetic evaluation. The underlying genetic defect was initially unknown. The family was enrolled in a study that sought to identify the underlying genetic defect and to describe the genotype and phenotype of FTAAD.

In this family (Fig. 1), two individuals (I:1 and I:2) had died from a thoracic aortic dissection or rupture and two individuals (II:2 and II:3) had survived an acute type A thoracic aortic dissection. One individual (II:8) had an intramural hematoma in the descending aorta. One family member (III:6) died due to an aortic rupture at the age of 23 years, after enrollment in this study but before any investigations were performed. After genetic counseling, all family members agreed to undergo clinical examination and genetic testing.

The regional ethical review board at Umeå University approved this study. Signed informed consent was obtained from all participants.

### Sanger sequencing and exome sequencing

DNA was extracted from whole blood via the salting-out method using a standard protocol. We obtained DNA from formalin-fixed, paraffin-embedded biological material from one deceased individual (I:2); for an another individual (III:6), we used DNA extracted from a Guthrie card. Sanger sequencing was performed to exclude causative variants in *FBN1, COL3A1, TGFBR1, TGFBR2* and *ACTA2*.

Exome enrichment was performed using 3 μg of genomic DNA from three afflicted individuals (II:2, II:3, and II:8). DNA samples were sheared via sonication with the Covaris S2 instrument (Covaris, Woburn, MA, USA). Fragment libraries were constructed from the sheared samples with the AB Library Builder System (Life Technologies, Thermo Fisher Scientific, Waltham, MA, USA) and target enrichment was performed with the Agilent SureSelect Human All Exon v4 kit according to the manufacturer’s instructions (Agilent Technologies, Santa Clara, CA, USA). This kit includes exonic sequences from 20,965 genes (corresponding to a total of 334,378 exons) that cover 51 Mb of genomic sequence (as specified by the company). Exome capture was conducted by hybridizing the DNA libraries with biotinylated RNA baits for 24 h, followed by extraction with streptavidin-coated magnetic beads. Captured DNA was amplified and emulsion PCR was carried out with the EZ Bead System (Life Technologies). Sequencing on the SOLiD 5500xl System generated >40 million reads of length 75 bp for each sample. Individual libraries were labeled via a post-hybridization barcoding procedure (Agilent Technologies; barcodes were compatible with SOLiD sequencing technology).

### Analysis of sequencing data

Alignment of color-space reads to the human reference genome (hg19) was performed using Lifescope Genomic Analysis Software v2.1 (Life Technologies). Approximately 90 % of the reads from each sample were uniquely mapped to the target regions, generating an average coverage of 35X and a median coverage of 30X over the targeted exons across samples. Single-nucleotide variants (SNVs) and small insertions and deletions (indels) were subsequently called by the diBayes algorithm available within Lifescope. All called SNVs and indels were imported into a local installation of the CanvasDB database system for annotation and further analysis [8].

First, filtering was performed to identify variants that were present in samples II:2, II:3, and II:8 but not in any of the >1000 exomes present in our local CanvasDB exome database [8]. To account for deviant clinical situation of individual II:8, we also filtered the data to identify variants shared between the affected individuals II:2 and II:3 but not present in individual II:8 or in any of the sequenced individuals in CanvasDB.

Sanger sequencing was performed on exon 17 of the *MYLK* (NM_053025.3, ENST00000360304) in affected family members to verify the presence of the identified *MYLK* deletion. We performed segregation analysis of the deletion in both affected and unaffected family members.

### cDNA analysis

Total RNA was extracted from the whole blood of two carriers (II:2 and II:3) and two non-carriers (II:4 and II:5) using Trizol (Life Technologies) and then converted into cDNA by using RevertAid H Minus First Strand cDNA Synthesis Kit (Fermentas, Thermo Fisher Scientific). Bidirectional Sanger sequencing (Applied Biosystems BigDye Terminator v3.1 Cycle Sequencing Kit, Applied Biosystems, Thermo Fisher Scientific) on a 3730xl DNA Analyzer (Applied Biosystems) was used for analysis of *MYLK* cDNA (NM_053025.3 ENST00000360304) using primers in exon 17 (ENSE00003507438) and 18 (ENSE00003603809) to confirm the mutation or to verify nonsense-mediated decay. Sequence analysis was performed using the Sequencher (Gene Codes Corporation, Ann Arbor, MI, USA). Primers sequences where designed using Primer 3 Plus [9] and are available upon request. Prediction of the effect of sequence variants on protein function was performed using MutationTaster [10].

### Clinical examination

Detailed medical histories, including diseases and medication, were obtained for each family member. Clinical examination was conducted in order to exclude known syndromic forms of FTAAD and to identify phenotypic characteristics common to family members. Height and weight were measured using a calibrated stadiometer and scale. Body surface area (BSA) was calculated according to the formula by DuBois and DuBois [11]. Joint mobility was assessed according to the Beighton hypermobility score. Twelve-lead electrocardiography was performed.

### Transthoracic echocardiography

Transthoracic echocardiography was performed using a Vivid 7 (GE Medical Systems, Horten, Norway) echocardiography machine. Aortic diameter was measured from the parasternal long-axis view at the sinuses of Valsalva (SoV) and at the widest level of the ascending aorta (AoA). All measurements were made in end-diastole and considered the inner edge-to-inner edge distance from the parasternal long-axis view. Measurements were made in M-mode after verifying correct positioning and alignment in the two-dimensional image. For analysis of aortic distensibility, the maximal systolic diameter was measured in M-mode at the same level in the ascending aorta. The average of three measurements in different cardiac cycles was calculated. Diameters were indexed to age, sex and body surface are. Aortic distensibility was calculated as (AoAmax–AoAmin)/(AoAmin × PP), where AoAmax is the ascending aortic diameter in systole, AoAmin is the ascending aortic diameter in diastole, and PP is the pulse pressure (systole minus diastole) calculated from the blood pressure measured in the left arm at the end of the echocardiographic examination. All examinations were performed by one of two sonographers, then reviewed and analyzed offline by one experienced investigator. Data published by Mirea et al. were used as reference echocardiographic diameters [12].

### Magnetic resonance imaging (MRI)

MRI was performed with the Achieva 3.0 T MRI system (Philips, Best, The Netherlands) with the patient in the supine position. All imaging was electrocardiography gated with a three-lead vector electrocardiogram and acquired during expiratory breath hold. Localizer sequences were followed by transaxial T1- and T2-weighted “black blood” sequences over the heart and the great vessels. The internal diameters of the ascending and descending aorta were measured at the level of the pulmonary bifurcation by a single reader without knowledge of the diameters obtained via echocardiography. Data from Davis et al. [13] were used as reference values for MRI measurements of the ascending and descending aorta.

Cross-sectional lumen areas of the ascending aorta were determined throughout the cardiac cycle using a semi-automated contouring method (Segment® v1.9, MedViso, Lund, Sweden) at the level of pulmonary bifurcation and edited when needed [14]. All tracings were reviewed and edited by one observer. Systolic and diastolic arterial blood pressures were measured in the beginning of the flow sequences. Aortic distensibility was calculated with the formula D = (AoAmax–AoAmin)/(AoAmin × PP), where AoAmax is the largest area in ascending aorta (systole), AoAmin is the smallest area (diastole) and PP is the pulse pressure.

### Arterial stiffness

Arterial stiffness was measured with Arteriograph® (TensioMed, Budapest, Hungary) after the subject rested for 5 min in the supine position. Arteriograph® enables pulse wave analysis via an oscillometric method [15]. Pulse wave velocity (PWV) was used as a marker of aortic stiffness. The method has been validated against invasive and other non-invasive methods to measure PWV [16–18].

### Other examinations

Mutation carriers underwent computed tomography (CT) angiography of the aorta, main aortic branch vessels, and the arteries of the brain. One radiologist at Umeå University Hospital reviewed these evaluations.

### Statistical analysis

Continuous variables are presented as means and standard deviations. The non-parametric Mann-Whitney *U*-test was used to compare aortic diameter, distensibility, and PWV in mutation carriers and non-carriers. Linear regression models were used to study age-dependent progressions in aortic diameter, distensibility and PWV stratified for carriers and non-carriers. We estimated the 95 % confidence intervals for age-dependent changes in each group. All analyses were performed using SPSS version 22 (IBM, Armonk, NY, USA).

## Results

### Sequencing, exome sequencing and cDNA analysis

No known pathological sequence variants were identified in the *FBN1, COL3A1, TGFBR1, TGFBR2,* or *ACTA2* in affected individuals. When we filtered variants that were present in all three exome-sequenced afflicted individuals (II:2, II:3, and II:8) but absent from the >1000 exomes present in our local database, we identified sequence variants in *SEMA4A, PLCZ1, FBLN5, SPG11,* and *RANBP2*. None of these genes have been previously associated with FTAAD. The gene encoding fibulin 5 (*FBLN5*) contributes elastogenesis and vascular development and it is highly expressed in developing arteries [19]. We therefore performed segregation analysis for the *FBLN5* sequence variant in multiple affected family members, but found no co-segregation with the familial disease.

We also performed filtering to identify sequence variants common to the two brothers (II:2 and II:3) but not present in the female second cousin (II:8) or in any of the exomes in our local exome database. This process revealed 25 sequence variants, including a 2-bp deletion in *MYLK* (c.3272_3273del). Pathogenic sequence variants in *MYLK* were previously reported to cause FTAAD.[7] Variant c.3272_3273del results in a frame shift and a premature stop codon (p.Ser1091*). This deletion was not present in any of the exomes in our local database or in controls in Exome Sequencing Project or 1000 Genomes [8, 20, 21]. We identified co-segregation of this deletion with the disease in all affected family members except individual II:8 (Fig. 1). Thus, this sequence variant is pathogenic and disease-causing according to the American College of Medical Genetics evidence-based guidelines [22].

**Fig. 2:**
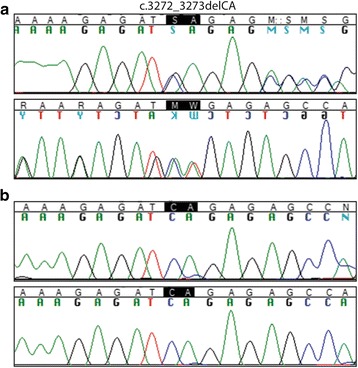
Variant sequencing. **a** Sanger sequencing of exon 17 in a heterozygous carrier (II:2) verified the presence of the c.3272_3273delCA variant in *MYLK* and the resulting frameshift. Sequencing was performed using a forward (*top*) and a reverse (*bottom*) primer. **b** Amplification of *MYLK* from cDNA using primers towards exon 17 and 18 identified only normal sequence in a homozygous non-carrier (II:2) (*top*) and in a heterozygous carrier (II:5) of the c.3272_3273delCA variant (*bottom*), thereby confirming nonsense-mediated mRNA decay.

Analysis with MutationTaster predicted that variant c3272_3273del would cause disease due to the introduction of a premature termination codon that would lead to nonsense-mediated mRNA decay. Sanger sequencing of cDNA from the two individuals carrying the *MYLK* variant (II:2 and II:3) detected only the normal sequence present in the two sequenced non-carriers (II:4 and II:5), thereby confirming nonsense-mediated mRNA decay of the mutant transcript (Fig. 2).

Nineteen individuals from this family had the opportunity to participate in this study. Eleven individuals were identified as carrying c.3272_3272del and eight individuals lacked the pathogenic sequence variant (Fig. 1). Three family members died due to aortic dissection (I:1, I:2, and III:6); biological material saved from individuals I:2 and III:6 indicated that both were carriers of this mutation. Clinical data for the five affected individuals identified as carriers of the *MYLK* mutation are presented in Table 1.

**Table 1:** Characteristics of family members with aortic event and presence of the 2-bp deletion in *MYLK*

ID	Gender	Age at event (years)	Dissection type	Entry	AoA Diameter (mm)	Survival	Surgery
I:1	F	70	Rupture	u/k	u/k	No	No
I:2	M	75	A	u/k	u/k	No	No
III:6	M	23	Rupture	AoA	u/k	No	No
II:3	M	43	A	AoA	47	Yes	Yes
II:2	M	55	A	AoA	48	Yes	Yes

*F* female, *M* male, *AoA* ascending aorta, *A* aortic dissection, type A, *u/k* unknown

### Previous aortic events, medical history, and physical examination

Five individuals in this family suffered aortic dissection or rupture of the ascending aorta and one individual had an intramural hematoma in the descending aorta. Of these six family members, three individuals died and three survived. Autopsy reports were obtained for all three cases.

In the autopsy report of individual I:1 (female, aged 70 years), the ascending aorta was described as ruptured with hemorrhage into the pericardial sac. There was no comment on the size of the ascending aorta. No biological sample from the autopsy was archived, prohibiting mutation analysis; however, we consider this individual to be an obligate carrier.

Individual I:2 (male, aged 75 years) was hospitalized due to acute chest pain that migrated to the abdomen and legs. He was referred for CT imaging but experienced sudden circulatory arrest before the imaging was performed. Autopsy revealed a dissection from the sinuses of Valsalva to the abdominal aorta as well as blood in the pericardial sac. The report contained no information about the size of the aorta. Four years earlier, at the age of 71 years, transthoracic echocardiography indicated that the diameter of his ascending aorta was 39 mm. Mutation analysis confirmed that he carried the 2-bp deletion in *MYLK*.

Individual III:6 (male, aged 23 years) was found deceased at home. Autopsy revealed an aortic rupture approximately 1.5 cm above the aortic valve in the posterior wall of the ascending aorta. There was blood in the pericardial sac. The rupture was localized to a protrusion of the aortic wall but the ascending aorta was not generally dilated. Mutation analysis for the *MYLK* deletion was positive. During MRI of the spinal cord 6 years earlier, the ascending aorta was measured as 28 mm in diameter.

Two individuals (II:2 and II:3) survived a type A dissection and underwent emergency surgery to place conduits in their ascending aorta. They were both carriers of the *MYLK* deletion.

Individual II:3 suffered an aortic dissection at the age of 43 years. He was previously healthy and was suddenly affected by chest pain. CT revealed a type A aortic dissection from the ascending aorta to the proximal descending aorta. The diameter of the dissected ascending aorta was 47 mm. He received a supracoronary graft, reconstruction of the aortic arch, and an elephant trunk. There were no further cardiovascular events during 13 years of follow up.

Individual II:2 underwent CT of the thoracic aorta, at the age of 48 years, after his brother’s (individual II:3) aortic dissection. The ascending aorta was 35 mm in diameter at that time; this was considered normal and no further follow-up was performed. Seven years later, at the age of 55 years, he suffered an aortic dissection. At that time, he was healthy and he was not taking any medication. CT imaging showed a type A aortic dissection from the ascending aorta to the bifurcation of the abdominal aorta. The diameter of the ascending aorta was 48 mm. The dissection did not involve the aortic root and he received a supracoronary graft. He has bronchial asthma, but otherwise he is doing well.

Individual II:8 harbored an acute circumferential intramural hematoma in the descending aorta at the age of 59 years. There was no aortic dissection and she was treated conservatively. One month later the wall thickening of the descending aorta was normalized and the diameter of the descending aorta was normal. There was no medical history of aortic events in her parents or grandparents. Exome sequencing did not identify the *MYLK* mutation in this individual but a sequence variant in *FBN5* was present.

No one of the carriers had experienced abnormal bleeding or wound healing after minor operations. Their surgical scars appeared normal. None of the women described bleeding or other problems during the pregnancy, delivery or post partum. There were no common symptoms from the gastrointestinal tract, urinary tract or circulatory system. No musculoskeletal, joint, or eye symptoms were evident. Four carriers have bronchial asthma, one has persistent atrial fibrillation and one has osteoarthrosis of the wrists.

Physical examination did not reveal abnormalities common to carriers. They had normal body constitution and there was no joint hypermobility (Beighton hypermobility score).

Electrocardiography was normal for all the individuals except for two. One individual displayed with atrial fibrillation and the other exhibited incomplete right bundle branch block.

### Aortic diameter and stiffness

**Fig. 3:**
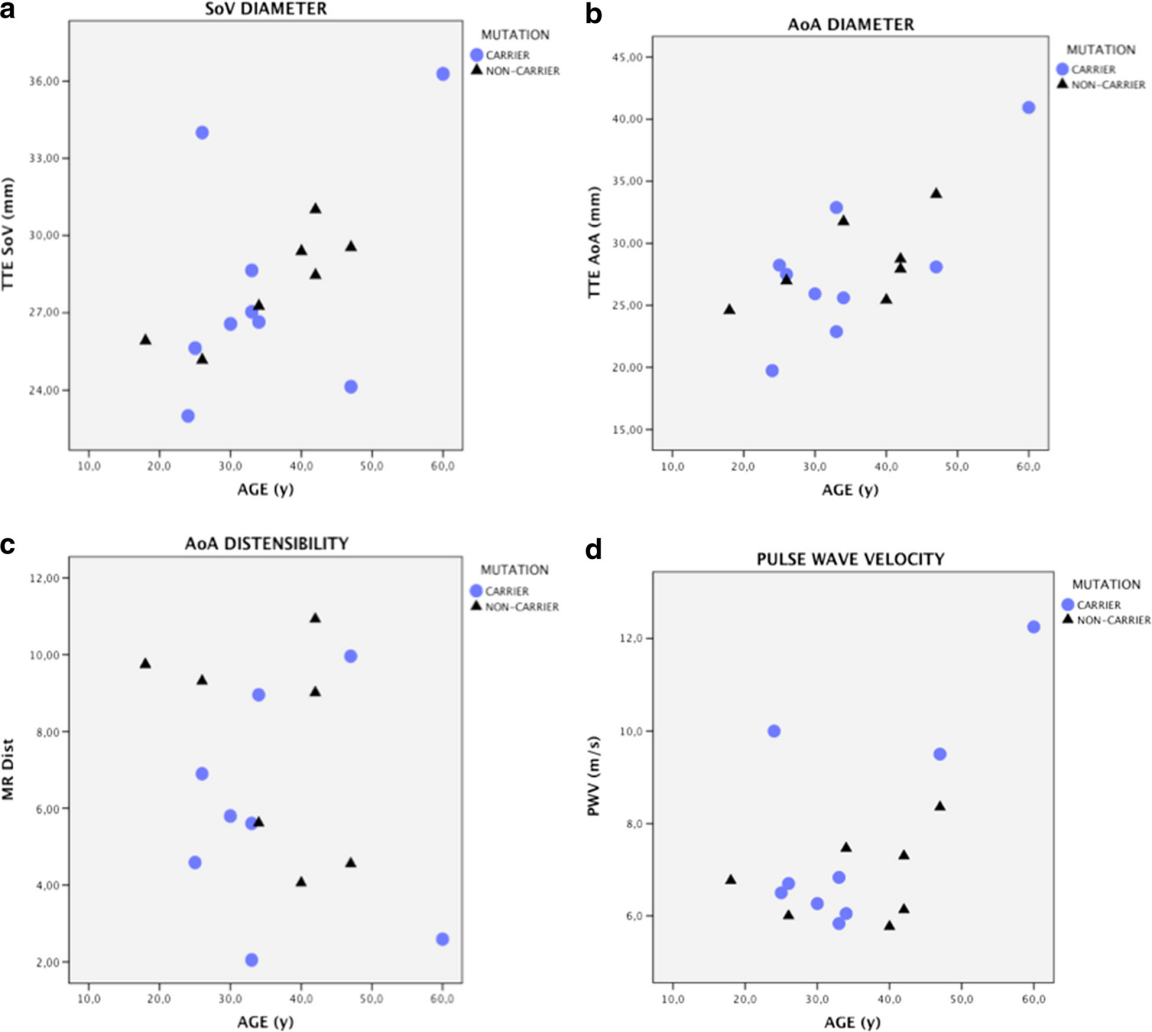
Diameter of the sinuses of Valsalva in mm (**a**), diameter of the ascending aorta (**b**), AoA distensibility in 10^−3^mmHg^−1^ (**c**), and PWV in m/s (**d**) in mutation carriers and non-carriers. MR = magentic resonance imaging, TTE = transthoracic echocardiography, SoV = sinuses of Valsalvae (mm), AoA = ascending aorta (mm), Dist = distensibility, PWV = pulse wave velocity

None of the carriers had dilated ascending or descending aortas (Fig. 3). Only one individual (II:4), a non-carrier, had a slightly dilated ascending aorta according to the reference values. The aorta measured 34 mm in diameter (20.5 mm/m^2^) at the age of 47 years; her body surface area was rather small (1.66 m^2^).

One of the carriers (II:1) had an ascending aortic diameter near the upper normal limit. At the age of 60 years, the ascending aorta was 41 mm (19.9 mm/m^2^) in diameter at the widest part (on transthoracic echocardiography) and 38 mm in diameter at the level of the pulmonary artery bifurcation (on MRI). He clearly differed from the other individuals in terms of PWV (12.3 m/s) and aortic distensibility (2.59 10^−3^mmHg^−1^). This individual is the brother of the two individuals that suffered aortic dissections. After receiving information regarding prophylactic operation, including information about surgical risk, he agreed to undergo a prophylactic operation in 2014.

Statistical analyses uncovered no differences in aortic diameter, aortic stiffness, or PWV between carriers and non-carriers (Fig. 3). However, age-dependent developments in PWV appeared to differ between carriers and non-carriers, but did not reach statistical significance (Fig. 3d). The increase in PWV per year was estimated among non-carriers as 0.03 m/s (95 % CI −0.07–0.13) and among carriers as 0.13 m/s (95 % CI 0.00–0.30). This observation suggests an approximately four-fold higher age-dependent increase in PWV in carriers of c.3272_3273del versus non-carriers.

### Histology

In aortic specimens from three individuals with known dissections or ruptures (II:2, II:3, and III:6), histology revealed discontinuities in elastin fibers in the medial layer and surrounding the ruptured area (Verhoeff-van Gieson stain). No pathological findings were visible in the SMCs. Histopathological specimens from the individual who had prophylactic surgery (II:4), displayed no pathology. Previously described significant increase of arteries in the medial layer could not be seen in these specimens [7]. However, there were artifacts in all specimens and no immunohistochemistry was performed, which clearly compromises the possibility of determining the condition of SMCs in members of this family.

### Radiology

All carriers underwent CT angiography of the aorta, the main aortic branch vessels and the vessels of the head and neck. The two individuals with aortic dissection (II:2 and II:3) both displayed a small aneurysm (4 mm) in the right medial cerebral artery. No intracranial aneurysms were observed in the other carriers. Aortic branch vessels did not exhibit any obvious tortuosity in any carriers. One carrier had an arteria lusoria (right subclavian artery originating from the aortic arch), which can be regarded as a normal variant. Two carriers had a mild stenosis of the celiac artery with post-stenotic dilation, but no other vascular abnormalities. Two other carriers had increased numbers of mesenterial lymphnodes. One of these individuals displayed thickening of the duodenal and jejunal wall, but had not experienced any gastrointestinal symptoms.

## Discussion

The 2-bp deletion in *MYLK* (c.3272_3273del) identified in the family investigated here leads to a premature stop codon and a nonsense-mediated decay of the transcript (Fig. 2). This decay should in turn result in insufficient kinase activity and possible SMC dysfunction in the aortic wall. Due to the decreased SMC contraction, the wall of the ascending aorta may not withstand the biomechanical forces from the pulsatile blood flow and may thus be at risk for rupture or dissection.

Arterial walls stiffen with age. The major changes are luminal enlargement and a reduction in the elastic properties of the arterial wall, mainly at the level of large elastic arteries. Patients with Marfan syndrome exhibit reduced elasticity in the ascending aorta and increased PWV in the arterial tree [23–25]. Reduced distensibility and increased PWV have also been observed in *MYH11* mutation carriers [26]. Therefore, SMC dysfunction due to *MYLK* mutation may lead to prematurely decreased distensibility of the large arteries and hence to increased PWV.

The present study identified no differences in aortic diameter or aortic stiffness between carriers of c.3272_3273del and non-carriers, but a tendency to higher age-dependent increase in PWV was observed in carriers compared with non-carriers. Some of the carriers were associated with notably low distensibility or high PWV for their age. However, the two groups were small, and conclusions must be drawn with caution.

Phenotype varied among carriers. Dissections occurred between 23 years and 77 years of age. At the time of dissection, the diameter of the ascending aorta varied from near- normal to dilated. The two individuals that survived aortic dissection (II:2 and II:3) had ascending aorta diameters of 47 mm and 48 mm when they dissected. The diameter of the aorta is known to increase acutely due to the dissection; this increase is most pronounced in the ascending aorta and can be as much as 13 mm [27]. Therefore, the diameters of the ascending aorta in these two individuals may have been considerably smaller at the time of dissection. These two individuals each harbored a small aneurysm in the medial cerebral artery. No intracerebral aneurysms or other aneurysms were evident in the other members of this family. There was no obvious arterial tortuosity of the arterial tree in any carriers. Four of the carriers had asthma. Otherwise, there were no other common symptoms or findings in carriers of variant c.3272_3273del.

Mutations in *MYLK* cause a vascular disease that is different both from that in Marfan syndrome and from that associated with mutations that disrupt TGF-β signaling pathways (mutations in *TGFβR1, TGFβR2, SMAD3, TGFβ2, TGFβ3*). In addition to aortic root enlargement, patients with Marfan syndrome display other syndromic features and are often identified before an aortic complication occurs. A subset of mutations in the TGF-β signaling pathway cause Loeys-Dietz syndrome, with varying degrees of clinical features and vascular disease [28].

Mutations in *ACTA2* and *MYH11* disrupt proteins involved in SMC contraction and result in predisposition to aortic aneurysms and dissections. Carriers of mutations in *ACTA2* gene often present with aortic events and in the absence of other clinical features, a situation similar to that experienced by carriers of *MYLK* mutations [29]. Familial occurrence of aortic dissections should always be investigated even in the absence of syndromic clinical features.

Preventing aortic dissection in MYLK mutation carriers remains a clinical challenge. As demonstrated in the current investigation, diameter alone is not a reliable marker for evaluating the risk of rupture or dissection. Here, there was no obvious difference in aortic stiffness between carriers of c.3272_3273del and non-carriers, at least at younger ages. Long-term follow-up of carriers and non-carriers may clarify the role of aortic stiffness in these patients. The importance of further genetic studies of FTAAD cannot be overemphasized. Identification of the underlying genetic defect will clarify the pathophysiology of aortic dissection and help avoid unjustified investigations and unnecessary concerns in non-carriers, with a concomitant shift of emphasis to carriers.

## Conclusions

Individuals carrying this deletion in *MYLK* have a high risk of presenting with an acute aortic dissection or rupture. Aortic events occur over a wide range of ages and are not always preceded by obvious aortic dilatation. Aortic elastic properties do not differ between carriers and non-carriers of this mutation, rendering it uncertain whether and when carriers should undergo elective prophylactic surgery.

Sequencing of the known gene variants causing aortic dissections should be done in all FTAAD patients. Identification of the causative gene allows identification of additional family members at risk for aortic disease and gene-based specific management of the carriers.

## Abbreviations

AoA: Ascending aorta
BSA: Body surface area
FTAAD: Familial thoracic aortic aneurysms and dissections
MYLK: Myosin light chain kinase
PWV: Pulse wave velocity
SMCs: Smooth muscle cells
SNVs: Single-nucleotide variants
SoV: Sinuses of Valsalva
